# 

**Published:** 1923-07

**Authors:** 


University of Bristol.—Students of the University have recently passed the following examinations :—

F.R.C.S. Eng.—W. Salisbury.

M.B., Ch.B. Bristol.—*Second Examination :* J. F. O. Bodman, A. H. Bright, A. J. M. Grimston, F. W. T. Hughes. *Final Examination* (*2nd Class Honours*) *:* F. H. Bodman, Constance L. Griffiths. *Pass :* D. Staley, I. Williams, *Part I.* (*including Forensic Medicine*) *:* E. R. Clutterbuck, E. B. Eedle, J. A. James, F. G. Jenkins, F. Langford, G. S. Mundy, A. S. Prowse. *Part I. only :* R. H. Dummett, K. F. Platt. *Forensic Medicine only :* Helen M. Dixon.

Conjoint Examining Board.—*Medicine :* W. Gornall, A. E. Sherwell, G. Murray-Shirreff, E. G. Bradbeer, *Carrie H. Osmond, Hilda R. Dutton. *Surgery :* Carrie H. Osmond, J. R. Nicholson-Lailer, H. M. Golding, *W. A. Gornall, C. Powell. *Midwifery :* Edna V. Butler, C. F. Wilson, J. R. Nicholson-Lailey, R. C. Hatcher, E. G. Bradbeer, H. R. Dutton, W. A. Gornall, J. L. Griffin, A. G. Heron, Doris E. Joscelyne, E. F. King, A. E. Sherwell.

L.D.S., R.C.S. Eng.—D. J. Andrews.

L.D.S. Bristol.—*Second Examination :* W. B. Bowrey, A. W. Cawsey, C. S. Cossham, R. G. Downes, D. Griffiths, C. H. Hill, H. F. Lane, S. Phillips, B. F. Robinson, Agnes M. Thoseby, G. C. Brooks. *Anatomy only :* C. P. Winstone. *Third Examination :* G. C. Friend. *Final Examination :* V. E. N. Allen, H. N. V. Barnes, S. H. Bedford, A. S. C. Boulter, M. P. Browning, A. H. Butcher, R. G. Butterworth, H. P. Evans, G. Holt, A. R. Hutchings, C. Lewis, F. C. G. Lewis, J. C. W. Lewis, R. C. Price, J. F. Rigg, J. S. Roberts, W. H. Simpkiss, C. E. Smith, E. K. Tratman, Kathleen M. Watson, F. C. Winter.

L.M.S.S.A.—M. M. Lopez.

* Qualified.

